# The neural crest lineage as a driver of disease heterogeneity in Tuberous Sclerosis Complex and Lymphangioleiomyomatosis

**DOI:** 10.3389/fcell.2014.00069

**Published:** 2014-11-25

**Authors:** Sean P. Delaney, Lisa M. Julian, William L. Stanford

**Affiliations:** ^1^Sprott Centre for Stem Cell Research, Regenerative Medicine Program, Ottawa Hospital Research InstituteOttawa, ON, Canada; ^2^Faculty of Graduate and Postdoctoral Studies, University of OttawaOttawa, ON, Canada; ^3^Department of Cellular and Molecular Medicine, University of OttawaOttawa, ON, Canada; ^4^Department of Biochemistry, Microbiology, and Immunology, University of OttawaOttawa, ON, Canada

**Keywords:** Lymphangioleiomyomatosis, Tuberous Sclerosis, neural crest, cell of origin, disease modeling

## Abstract

Lymphangioleiomyomatosis (LAM) is a rare neoplastic disease, best characterized by the formation of proliferative nodules that express smooth muscle and melanocytic antigens within the lung parenchyma, leading to progressive destruction of lung tissue and function. The pathological basis of LAM is associated with Tuberous Sclerosis Complex (TSC), a multi-system disorder marked by low-grade tumors in the brain, kidneys, heart, eyes, lung and skin, arising from inherited or spontaneous germ-line mutations in either of the *TSC1* or *TSC2* genes. LAM can develop either in a patient with TSC (TSC-LAM) or spontaneously (S-LAM), and it is clear that the majority of LAM lesions of both forms are characterized by an inactivating mutation in either *TSC1* or *TSC2*, as in TSC. Despite this genetic commonality, there is considerable heterogeneity in the tumor spectrum of TSC and LAM patients, the basis for which is currently unknown. There is extensive clinical evidence to suggest that the cell of origin for LAM, as well as many of the TSC-associated tumors, is a neural crest cell, a highly migratory cell type with extensive multi-lineage potential. Here we explore the hypothesis that the types of tumors that develop and the tissues that are affected in TSC and LAM are dictated by the developmental timing of *TSC* gene mutations, which determines the identities of the affected cell types and the size of downstream populations that acquire a mutation. We further discuss the evidence to support a neural crest origin for LAM and TSC tumors, and propose approaches for generating humanized models of TSC and LAM that will allow cell of origin theories to be experimentally tested. Identifying the cell of origin and developing appropriate humanized models is necessary to truly understand LAM and TSC pathology and to establish effective and long-lasting therapeutic approaches for these patients.

## Introduction

### Clinical features in TSC and LAM reveal a heterogeneous disease spectrum

**Figure 1:**
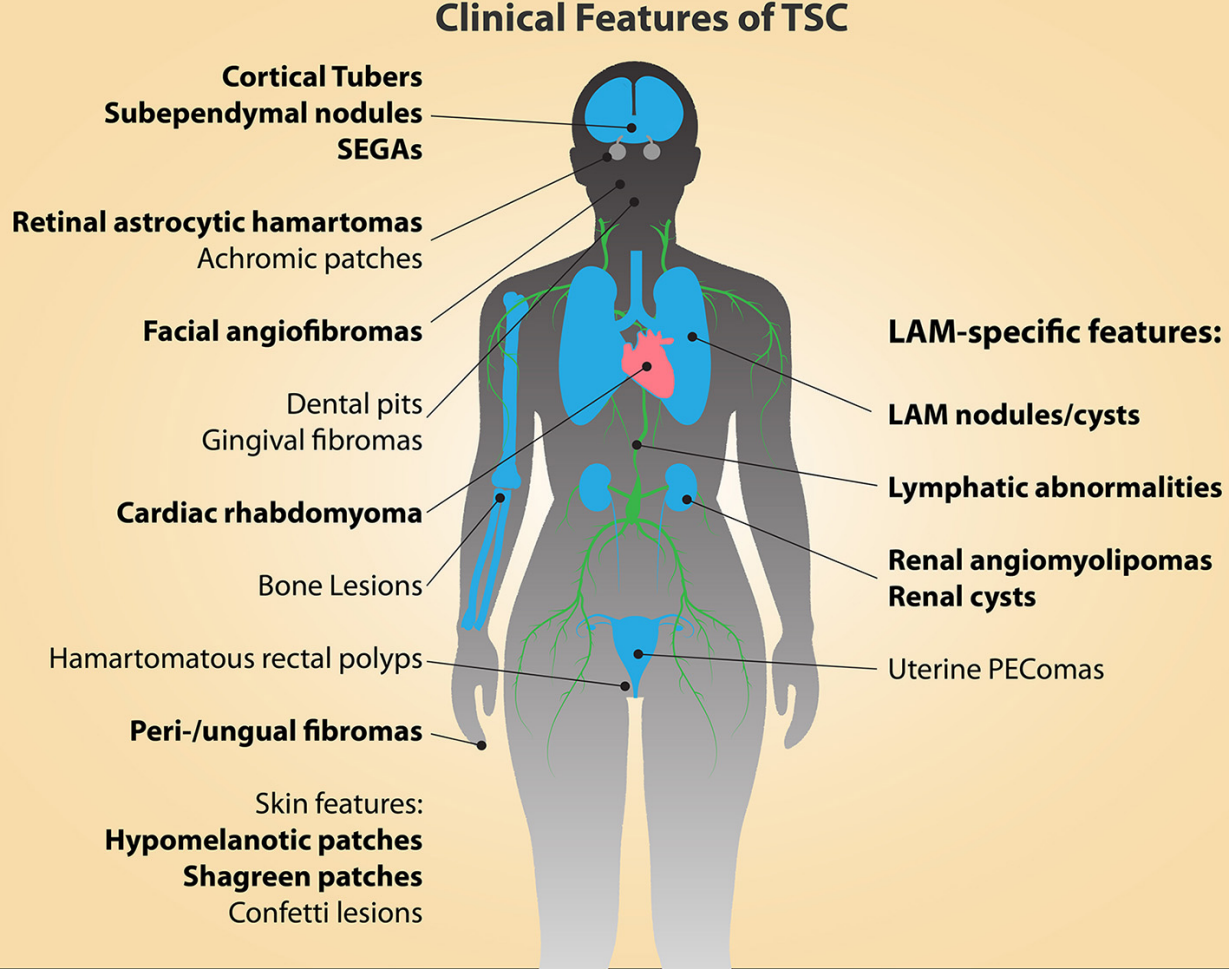
**The clinical manifestations of TSC and LAM are diverse and affect multiple organs and tissues**. The major diagnostic features of TSC are indicated in bold type (Northrup et al., 2013). Cortical tubers and cardiac rhabdomyomas occur during fetal development. Facial angiofibromas, hypomelanic macules, and retinal astrocytic hamartomas can be detected in infancy, while the other features continue to present themselves throughout development into adulthood.

Tuberous sclerosis complex (TSC) is a multisystem disorder that arises as a consequence of inherited or spontaneously acquired mutations in either the *TSC1* or *TSC2* gene. Along with Neurofibromatosis type 1, TSC is among the most common neurocutaneous diseases, occurring in an estimated 1 in 6000 births (Kandt, 2003; Kristof and Moss, 2011). TSC affects both children and adults, often with clinical manifestations initiating during embryonic development, accompanied by the gradual presentation of additional symptoms throughout childhood and into adulthood. Clinical features of TSC (Figure 1) include the appearance of low-grade tumors and malformations in the brain, heart, lungs, kidneys, eyes, skin, and bone, and loss of heterozygosity or second-hit mutations of the wild-type *TSC1* or *TSC2* allele are thought to be responsible for the formation of most of these lesions (Henske et al., 1996; Carsillo et al., 2000; Crino et al., 2010; Qin et al., 2010a, 2011; Tyburczy et al., 2014). Cortical tubers are prevalent in TSC and account for the majority of the debilitative neurological symptoms, including epilepsy, mental retardation and autism (Webb et al., 1996; Goh et al., 2005; Crino et al., 2006; Wong, 2006; Crino and Tsai, 2012). Tubers, along with cardiac rhabdomyomas, are formed during embryogenesis and can be detected prenatally (Park et al., 1997), whereas other tumors and lesions form during childhood and into adulthood. These include retinal astrocytomas, subependymal giant cell astrocytomas (SEGAs), angiofibromas and hypomelanotic macules in the skin, sub-ependymal nodules/giant-cell tumors, and renal angiomyolipomas (AMLs).

TSC is also associated with pulmonary and lymphatic manifestations in the form of Lymphangioleiomyomatosis (LAM), a progressive neoplasm of the lung that occurs in at least 30% of women with TSC (TSC-LAM), with an average age of diagnosis of about 35 years (Henske and McCormack, 2012). LAM is characterized by the presence of multiple neoplastic nodules within the lung interstitium, composed of proliferating smooth muscle-like cells and abnormally large epithelial cells that express melanocytic markers. The proliferative LAM nodules form cystic lesions within the lungs, which lead to the destruction of the parenchyma, resulting in progressive shortness of breath, chylous pleural effusions, pneumothorax, and eventual respiratory failure (Kitaichi et al., 1995; Chu et al., 1999; Urban et al., 1999).

LAM can also occur in a sporadic form (S-LAM), where lung lesions and associated renal and lymphatic manifestations are similar to those observed in TSC patients, but other TSC-associated tumors are absent (Costello et al., 2000; Moss et al., 2001). In addition to pulmonary manifestations, other common features of LAM include lymphatic abnormalities, such as lymphadenopathy (Chu et al., 1999; Urban et al., 1999), renal AMLs, and uterine PEComas (Perivascular Epitheloid Cell tumors). AMLs are benign growths composed of adipocytes and, much like their pulmonary counterparts, smooth muscle cells, and are asymptomatic in most cases (Bissler and Kingswood, 2004). Uterine PEComas are, curiously, also characterized by cells of an epithelial morphology that express smooth muscle and melanocytic markers, similar to the cells that comprise LAM lung nodules (Martignoni et al., 2007; Hayashi et al., 2011; Henske and McCormack, 2012). Thus, TSC is associated with the development of a broad spectrum of pathological lesions, affecting a wide diversity of tissues and cell types.

### Genetic features in LAM and TSC

TSC, TSC-LAM, and S-LAM patients typically possess inactivating mutations in *TSC1* or, much more commonly, *TSC2* (Smolarek et al., 1998; Carsillo et al., 2000; Sato et al., 2002). Loss of heterozygosity in either of these genes from second-hit mutations is thought to be important in driving tumor development (Green et al., 1994; Sepp et al., 1996; Niida et al., 2001; Han et al., 2004; Henske and McCormack, 2012). According to the Leiden Open Variation Database (Fokkema et al., 2011), as of 2014 there are over 680 and 1500 reported unique pathogenic DNA variants in *TSC1* and *TSC2*, respectively. The wide array of mutations spans all exons of both *TSC1* and *TSC2*, with no specificity for any particular coding region or mutation type, and includes a number of mutation types [such as genomic deletions, missense and non-sense mutations and protein truncations (Cheadle et al., 2000; Crino et al., 2006; Au et al., 2007)]. Although mutations in either of these genes lead to the development of disease features, extensive genotype-phenotype analysis has been performed and found that mutations in *TSC2* are associated with more severe phenotypes than are mutations in TSC1 (Niida et al., 1999; van Slegtenhorst et al., 1999; Au et al., 2007).

Intriguingly, the genetic features in S-LAM patients mirror those of TSC patients, but pathological manifestations in S-LAM are limited to those in the lung, kidneys, lymphatics, liver and uterus (Henske and McCormack, 2012), and *TSC* gene mutations in S-LAM patients are not wide-spread or found within the germline (Astrinidis et al., 2000). Currently, the conditions that underlie the heterogeneity in the disease spectrum between TSC and LAM patients are unknown. Clinical observations, and some experimental evidence, offer clues, however, that the cell of origin in concert with the developmental timing of *TSC1* or *TSC2* mutation are likely key factors in determining which disease features ultimately manifest.

### Molecular characteristics of TSC and LAM lesions

#### Aberrant mTOR signaling broadly characterizes TSC lesions

TSC and LAM lesions are believed to share the common feature of elevated mTOR pathway signaling, ultimately as a consequence of inactivating mutations in *TSC1* or *TSC2* (Green et al., 1994; Niida et al., 2001; Han et al., 2004; Henske and McCormack, 2012). The *TSC1* and *TSC2* genes encode Hamartin and Tuberin, respectively, which form heterodimers and inhibit the GTPase activity of the Ras homolog Rheb, a direct activator of the Mammalian Target of Rapamycin (mTOR). mTOR signals through two macromolecular complexes, mTORC1 and mTORC2, to integrate the external environment (growth factor stimulation, cellular stress, oxygen availability, energy and nutrient levels) with diverse cellular functions, including proliferation, survival, growth and size, death and autophagy, protein translation, angiogenesis, movement, and migration. *TSC1* and *TSC2* function as critical gatekeepers of this multi-functional cellular signaling network.

The therapeutic efficacy of Rapamycin, a molecular inhibitor of mTOR, in treating many of the manifestations in TSC and LAM (Franz et al., 2006; Bissler et al., 2008; Krueger et al., 2010; Micozkadioglu et al., 2010; Casanova et al., 2011; Dabora et al., 2011; DeKlotz et al., 2011; McCormack et al., 2011; Tiberio et al., 2011; Canpolat et al., 2014; Park et al., 2014) has established elevated mTOR signaling as a key feature driving these tumors. The cells that comprise LAM and TSC tumors reflect many features of hyper-active mTOR signaling: abnormally large or “giant” cells, altered morphology, aberrant migration, abnormal proliferation, increased cell survival and reduced autophagy are common features of many lesions (Crino, 2004; Crino and Tsai, 2012; Henske and McCormack, 2012). Increased angiogenesis is also a feature of many TSC lesions, including LAM (Nguyen-Vu et al., 2001; Arbiser et al., 2002; Kumasaka et al., 2004; Li et al., 2013).

#### Unique molecular characteristics distinguish TSC-associated lesions

Despite the common element of hyperactive mTOR signaling, the types of lesions that develop in TSC and LAM are diverse, in terms of both the tissues that are affected and the molecular characteristics of the aberrant cells. Furthermore, different types of TSC-associated tumors exhibit additional behavioral and signaling characteristics that are distinct from one another. For example, LAM cells are uniquely responsive to estrogen and prolactin signaling, and express these receptors on their surface (Terasaki et al., 2010; Gao et al., 2014)(reviewed in Yu and Henske, 2010). They also express markers of smooth muscle cells and melanocytes, cell types not normally found throughout the lung interstitium.

Alternatively, the aberrant cells that comprise cortical tubers and SEGAs in TSC patients express markers of early neuronal and glial cell differentiation (Lopes et al., 1996; Ess et al., 2005; Zhou et al., 2011) and recent mouse models have clarified that these tumors can result from mTOR-dependent promotion of aberrant neural progenitor cell (NPC) expansion and premature differentiation, accompanied by defective maturation and abnormal migration (Magri et al., 2011; Magri and Galli, 2013). Thus, in the TSC lesions confined to the CNS, mTOR hyper-activation leads to a direct reprogramming of the self-renewal and differentiation capacity of the cells endogenous to the tissue in which the lesions are found, while the etiology of mTOR hyper-activation to the appearance of LAM-associated lesions in the lung, kidneys, and uterus is much less clear. TSC and LAM are further differentiated by the time-point of disease presentation, where most TSC features typically present in infancy or during childhood and LAM presents in adulthood. These differences suggest that, despite their common genetic characteristic, distinct TSC and LAM lesions exhibit unique pathological mechanisms.

### Potential cells of origin for TSC and LAM

The cell(s) of origin for TSC and LAM lesions are largely unknown, however clinical observations and limited experimental evidence have offered some key clues. The extensive multi-system involvement in these diseases is remarkable, and while there is evidence for both common and independent second-hit *TSC1* or *TSC2* mutations across multiple tumors within a given individual (Henske et al., 1996; Smolarek et al., 1998; Carsillo et al., 2000; Karbowniczek et al., 2003; Tyburczy et al., 2014), a single first-hit mutation appears to characterize all of a patients' tumors. The lesions that commonly arise in TSC are generally comprised of immature cells that exhibit molecular and behavioral features of either neuronal (central nervous system (CNS) lesions) or mesenchymal differentiation. While embryonic neural stem cells (NSCs) appear to function as a cell of origin for the CNS manifestations in TSC (discussed further below), the mesenchymal neural crest lineage has been postulated to broadly give rise to other types of lesions in TSC and LAM.

With the potential involvement of both NSCs and the neural crest lineage in disease heterogeneity, the influence of the developmental time-point at which *TSC1* or *TSC2* mutations occur on disease severity has also been called into question. However, these theories have not been experimentally confirmed, and no animal or humanized model currently exists to directly test these suppositions. Here, we explore the evidence supporting the cell of origin hypotheses that have emerged from the TSC and LAM field, and, based on these theories, we further propose approaches for generating relevant humanized models of TSC and LAM that will enable the identification of improved therapeutic avenues and open the door for patient-centered approaches to treatment.

### Experimental evidence that neural stem cells drive CNS pathology in TSC

#### Mouse models of TSC gene deficiency in the CNS

Experimental approaches to identify the cell of origin in TSC have focused to date almost exclusively on validating the hypothesis that the CNS tumors and behavioral phenotypes are driven by *TSC1* or *TSC2* mutations in the NSC lineage. Since the CNS manifestations cause significant morbidity and are apparent at such a young age, this focus is not surprising. As mouse models of both TSC1- and TSC2-deficiency using gene targeting lead to early embryonic lethality, and mice heterozygous for either *TSC* gene do not develop TSC-related CNS pathologies (likely due to the absence of a second-hit mutation during embryonic development) (Kobayashi et al., 1999, 2001; Onda et al., 1999; Kwiatkowski et al., 2002), more sophisticated models have recently emerged to assess the effects of homozygous TSC gene mutation in select populations of NPCs and differentiating or differentiated neuronal cells (Uhlmann et al., 2002; Meikle et al., 2007; Ehninger et al., 2008; Way et al., 2009; Goto et al., 2011; Magri et al., 2011; Zeng et al., 2011; Zhou et al., 2011; Carson et al., 2012; Feliciano et al., 2012, 2011; Magri and Galli, 2013; Normand et al., 2013; Prabhakar et al., 2013). These models have included a variety of drug inducible and non-inducible conditional knockouts, as well as adenoviral-mediated approaches or *in utero* electroporation to induce gene knock-out stochastically. These approaches have allowed for targeted induction of TSC1- or TSC2-deficiency in distinct neural cell populations, limited by regional location, the size of the affected cell population, or the developmental time-point of induction.

Arising from these efforts, mouse models of TSC1- or TSC2-deficiency now exist that closely model the CNS manifestations of TSC that occur in human patients, including neuronal network dysfunction and seizure activity, sub-ependymal nodules that lead to the development of SEGAs and elements of cortical tubers, accompanied by abnormal neuronal migration, enhanced astrogliosis, enlarged cells and cortical lamination defects (Goto et al., 2011; Magri et al., 2011; Carson et al., 2012; Magri and Galli, 2013; Normand et al., 2013; Prabhakar et al., 2013). Importantly, hyper-activation of mTORC1 signaling has indeed been confirmed as causative in the majority of TSC-like phenotypes in these models, confirmed by extensive phenotypic rescue following post-natal Rapamycin treatment in many of these studies. Interestingly, these models have revealed that while abnormal, enlarged cells and nodule-like formations can be induced to some degree by *TSC* gene deletion in virtually all neuronal populations (progenitors and post-mitotic cells) and at different developmental stages (throughout embryogenesis and postnatally), neurological features such as seizure activity are only clearly reproduced by targeting NPCs and neurons prenatally, during peak stages of neurogenesis. Furthermore, the overall severity of CNS manifestations following *TSC* gene deletion at different stages occurs on a gradient scale, with the most severe and extensive phenotypes associated with mutations in NPC populations at the earliest stages of neurogenesis (in neuroepithelial cells and radial glia at E9.5 and E12.5, respectively). As appearance of the full spectrum of TSC-associated CNS lesions seems to require *TSC* gene mutation in early NPCs, these studies together provide solid evidence to support the hypothesis that a neural stem or progenitor cell is the cell of origin for the CNS manifestations in TSC.

#### The proportion of TSC1/ TSC2-deficient cells affects phenotypic severity in mouse models

Not only have the CNS mouse models revealed that *TSC* gene deficiency at different stages of neural development can lead to different types of manifestations, but also that the degree of mosaicism of TSC1- or TSC2-deficient cells correlates with phenotypic severity. The first key element in generating mouse models that closely recapitulate the TSC condition was induction of *TSC* gene deficiency in specified neuronal sub-populations, as opposed to homozygous mutations in ESCs. This demonstrated that while wide-spread homozygous TSC1- or TSC2-deficiency within the germline results in overly severe, lethal phenotypes, restricted *TSC* gene mutations within the neuronal lineage can be better tolerated but lead to TSC-like phenotypes in the brain. Again, mutation at late vs. early developmental stages, in which fewer downstream cells would be directly affected, leads to less severe phenotypes.

Some recent mouse models have directly demonstrated experimentally, within distinct cell populations, that the degree of mosaicism for *TSC* gene deficiency within neural cells also leads to a spectrum of phenotypes. Specifically, mutation of *TSC1* in a subset of thalamic progenitor cells caused substantially greater neurological dysfunction, indicative of TSC-like pathology, if the mutation was made at mid-gestation (E12.5) vs. late gestation (E18.5) in an embryonic mouse model (Normand et al., 2013), and experiments in which subsets of NPCs were knocked-out for *TSC1* in variable numbers using adenoviral-mediated Cre injection into the cerebral ventricles showed that the severity of resulting TSC-like pathology was dependent on the dose and serotype of the injected virus (Prabhakar et al., 2013). These models have provided strong evidence that the CNS lesions and neuropathologies associated with TSC can be driven by TSC1- or TSC2-deficiency and consequent mTOR deregulation in NPCs, and together demonstrate that the severity of the TSC-associated CNS phenotypes exist on a spectrum that is strongly influenced by the developmental timing and degree of mosaicism for *TSC1* or *TSC2*.

### Heterogeneity in disease spectrum suggests distinct cells of origin and potential mosaicism in TSC

A clear distinction between individuals with *TSC* gene mutations who acquire Tuberous Sclerosis vs. S-LAM is that the somatic mutations in *TSC1* or *TSC2* are more widespread in those with TSC, and are typically also present in the germline (Dabora et al., 2001; Strizheva et al., 2001). This was a substantial discovery toward our understanding of how S-LAM may develop in the absence of other TSC manifestations, and pushed forth the concept that TSC and LAM lesions may develop from different founding cell types, and thus that TSC gene mutations are restricted to a smaller population of cells in S-LAM patients. The fact that there is considerable heterogeneity in the clinical presentation and severity of the disease spectrum among both LAM and TSC patients (Henske and McCormack, 2012; Curatolo and Maria, 2013) and evidence for gonadal and/or somatic mosaicism in some TSC patients that can correlate with phenotypic variability (Rose et al., 1999; Verhoef et al., 1999; Qin et al., 2010b; Boronat et al., 2014a,b) lend further support to the concept that disease spectrum is significantly affected by the proportion of cells, and likely the identity and fate of those cells, that possess a *TSC* gene mutation.

It is also possible that distinct *TSC1* or *TSC2* mutations present in different patients may also contribute to disease heterogeneity by encoding TSC proteins with varying degrees of function. Indeed, hypomorphic mutations have been observed in some TSC patients, and experimental and clinical evidence suggest that these mutations result in reduced phenotypic severity (Khare et al., 2001; O'Connor et al., 2003; Nellist et al., 2005, 2008; Jansen et al., 2006; Pollizzi et al., 2009; Yuan et al., 2012; Fu and Ess, 2013). However, these cases appear to represent a minority of known TSC patients. Nevertheless, in cases where a hypomorphic *TSC* gene mutation is present, it is likely that this feature acts in conjunction with the identity and proportion of cells that carry the mutation to influence phenotypic severity.

The findings from the TSC CNS-related mouse models discussed above provide strong experimental support for the concept presented here. Although these studies were limited to the CNS manifestations, it is likely that this theme persists throughout the full spectrum of the disease and that in general a more restricted mutational burden will lead to the development of fewer types of lesions and fewer numbers of lesions. With this in mind, one could postulate that while *TSC* gene mutations in NSCs lead to the CNS lesions, mutations within the neural crest lineage are responsible for the development of most other TSC lesions, including those associated with LAM, and that variable mutational burden within the neural crest (NC) lineage leads to the greatest heterogeneity in TSC disease phenotypes.

### Evidence supporting a neural crest cell of origin for LAM and other TSC-associated lesions

#### Neural crest cell activity during embryogenesis

The neural crest is a transient population of migratory progenitor cells that emerge at the interface between the dorsal region of the neural plate and the non-neural ectoderm in embryonic development during neurulation. Although the migratory patterns of the NC can differ slightly from species to species, studies performed in model organisms, such as *Xenopus*, mouse, quail and chick embryos, have offered great insight into the contributions of the NC population to various tissues in the developing embryo. Although exact migratory patterning in humans is unknown, lineage tracing has revealed enough commonality between these model species to establish a paradigm for neural crest cell (NCC) migration and differentiation during human development. Through contact mediated interactions, NCCs emerge, undergo epithelial-to-mesenchymal transition (EMT), and migrate in specific succession and order in an anterior to posterior sequence (Theveneau and Mayor, 2012). Of note, both the neural plate and the surface ectoderm contribute cells to the NC population (Moury and Jacobson, 1990; Selleck and Bronner-Fraser, 1995). Once NCCs have emerged and delaminated, they follow distinct migratory patterns depending on their eventual fate, the majority of which are summarized in Table 1.

**Table 1:** **Neural crest cells can be categorized into four major subgroups, based on their respective migratory pattern and the tissues to which they contribute**.

**NC Subtype**	**Tissues**	**Cell types**	
Cranial	Craniofacial skeleton	Fibroblasts	Neurons
	Skin	Melanocytes	Glia
	Cornea	Adipocytes	Mesenchymal cells
	Connective tissue	Osteocytes	Myocytes
		Odontoblasts	Pericytes
		Chondrocytes	
Cardiac	Branchial arches	Myocytes	
	Cardiac septum	Pericytes	
	Parasympathetic cardiac ganglia	Neurons	
		Glia	
Vagal	Enteric nervous system	Neurons	
		Glia	
Trunk	Peripheral nervous system	Neurons	
	Skin	Glia	
	Adrenal medulla	Melanocytes	
		Chromafinn cells	

It should be noted that individual NCC multipotency varies between subgroups as well as within subgroups themselves.

NCCs can be generally categorized into four major subgroups based on their migratory cohort: cranial, cardiac, vagal and trunk. Cranial NCCs contribute to the tissues of the craniofacial region, differentiating into the widest array of cell types of the neural crest subgroups, including vascular smooth muscle cells and pericytes, chondrocytes, adipocytes, osteocytes, odontoblasts, melanocytes, connective tissue, and sensory and parasympathetic ganglia (D'Amico-Martel and Noden, 1983; Köntges and Lumsden, 1996; Etchevers et al., 2001; Billon et al., 2007; Grenier et al., 2009). Cardiac NCCs differentiate into parasympathetic cardiac ganglia, as well as smooth muscle of the branchial arches and the cardiac outflow tract, and form the aorticopulmonary septum (Kirby et al., 1983; Kirby and Stewart, 1983). Vagal NCCs primarily give rise to the neurons and glia of the enteric nervous system (Yntema and Hammond, 1954; Le Douarin and Teillet, 1973). Finally, trunk NCCs give rise to melanocytes and the neurons and glia of the peripheral nervous system (Raible and Ungos, 2006).

#### Potential role for neural crest subtypes in TSC disease heterogeneity

NCCs are able to differentiate into a wide array of cell types that contribute to many of the tissues associated with TSC tumors. Theoretically, distinct NC subtypes could be specifically associated with some of the multi-systemic tumors observed in TSC. For instance, the cranial NC may be implicated in the development of facial angiofibromas, dental pits, and retinal tumors. Indeed, cases of retinal lesions have been reported in connection with neurofibromatosis, a disease also thought to be of neural crest origin (Ishiko et al., 2006; Sachdeva et al., 2010). Cortical tubers could also potentially be attributed to the cranial neural crest, a theory that has previously been postulated (Hindman et al., 1997). Extending this theory, TSC1- or TSC2-deficient cardiac NC may be the cell of origin for cardiac rhabdomyomas and even pulmonary LAM lesions (due to the involvement in aorticopulmonary septum development), and dysfunctional trunk NC may be responsible for “confetti” skin lesions and hypomelanotic macules. Within this frame of thought, the determining factor in which tumor types ultimately form is perhaps both spatial and temporal. If the first-hit mutation occurs early on during embryogenesis within a cell of the developing neural plate or surface ectoderm before neurulation, the potential number of affected progeny capable of acquiring a second-hit mutation would be much greater and could potentially affect all NC subgroups, leading to a more severe TSC phenotype affecting multiple tissues. If the first-hit mutation occurs later in development, perhaps in an emerging NC subtype, the affected cell may only have influence on the specific subtype emerging from its respective anterior/posterior position along the developing neural tube. Such a scenario could explain the extreme differences in TSC disease spectrum that are observed in highly affected TSC patients vs. individuals with S-LAM.

#### Neural crest stem cells persist in adult tissues associated with TSC lesions

While tissue innervation by migrating NCCs is a fundamental process during embryonic development, NCCs with stem cell-like properties are now known to persist in a number of adult tissues. Just as tissues that are innervated by embryonic NCCs during development theoretically have the potential to give rise to LAM-like cells or cells that comprise other TSC mesenchymal lesions, these adult neural crest stem cells (NCSCs) are a particularly promising population as a potential LAM and TSC cell of origin. The tissues in which NCSCs reside in the adult are diverse, and include the gut, bone marrow, heart, dental mesenchyme, dorsal root ganglia, and cornea (reviewed in Dupin and Sommer, 2012). Intriguingly, a number of distinct multipotent stem cells with neural and/or NC differentiation potential have also been identified in the skin (Dupin and Sommer, 2012). As NCCs possess inherent migratory and transdifferentiation potential (discussed further below), and exhibit extensive interactions with their surrounding microenvironments that can instruct migratory, differentiation and invasiveness behaviors, many of these cell types could potentially serve as the cell of origin for TSC lesions (Takahashi et al., 2013).

The susceptibility of the skin to ultraviolet (UV) radiation implicates resident skin NCSCs as promising cell of origin candidates, as UV-induced damage could induce second-hit mutations. For example, skin-derived precursors (SKPs), multipotent NCSCs found in the dermal sheath and papillae of hair follicles in both rodents and humans (Toma et al., 2001, 2005; Fernandes et al., 2004; Biernaskie et al., 2009; Jinno et al., 2010), have been heavily implicated as the cell of origin for the dermal tumors associated with the neurocristopathic disorder Neurofibromatosis type I (Le et al., 2009). A recent study has also provided compelling evidence that precursor cells found within the skin may serve as the cell of origin for facial angiofibromas in TSC patients, following the acquisition of UV-induced second-hit *TSC2* mutations (Tyburczy et al., 2014). Given this potential, it is a distinct possibility that SKPs may similarly serve as the cell of origin for LAM and the cells that comprise other TSC-associated lesions, providing that SKPs possessing *TSC* gene mutations are able to invade the lymphatic and/or cardiovascular circulation.

#### Clinical evidence suggests a neural crest origin for TSC and LAM lesions

Sequencing of tissue from LAM nodules or disseminated LAM cells from body fluids has indicated that germline *TSC1* or *TSC2* mutations are not prevalent in S-LAM patients (Astrinidis et al., 2000; Carsillo et al., 2000). Thus, the first-hit mutation that drives LAM must typically arise *de novo* in a somatic cell. The lack of CNS involvement in the pathology of S-LAM provides strong clinical evidence that the neural stem cell lineage is not affected in S-LAM patients. The molecular markers present in LAM cells within its classical lesions [alpha-SMA, HMB-45, TRP-1, MART-1, GD3 (Carbone, 2009; Gilbert et al., 2013)], as well as the tissue types affected in the majority of TSC-associated tumors, do however present significant clues to the potential cell(s) of origin. The tissues in which lesions/abnormalities are observed in TSC patients are diverse, but all of them share the common feature that they are wholly or in part comprised of neural crest progeny (Figure 1, Table 1) (Dupin and Sommer, 2012). These tissues include the heart (cardiac rhabdomyomas), the skin (angiofibromas, most predominant in the facial region), dental enamel, eyes (retinal astrocytic hamartomas), and the innervation of the kidneys (AMLs), uterus and lungs (Curatolo and Maria, 2013). Additionally, the abnormal cells observed in LAM lesions express molecular markers of cells that are generated by the neural crest; these include smooth muscle, melanocytes, and adipocytes.

In addition to key molecular markers, there is also compelling evidence that TSC and LAM cells are able to migrate between different tissue sites (Smolarek et al., 1998; Karbowniczek et al., 2003), reminiscent of the highly migratory behavior of neural crest cells. Furthermore, LAM lung lesions contain a heterogeneous population of cells, with both highly proliferative spindle shaped cells and large epithelial cells making up LAM clusters. Moreover, both of these cell types express molecular markers of mesenchymal neural crest cells *in vivo* (e.g., smooth muscle actin, and HMB45 or gp100 in the epithelial cells). This suggests the intriguing possibility that, if indeed LAM cells originate from the neural crest, they may possess the ability to undergo EMT or even mesenchymal-to-epithelial transition (MET) within the lesion itself, accounting for the presence of these distinct cell types within single lesions.

#### LAM cells exhibit extensive migration and EMT

Just as neural crest cells represent a highly migratory cell population, a striking characteristic of LAM cells is their ability to travel from one tissue site to another, leading to multi-tissue tumor formation. For example, mutation analysis has revealed that the same mutations are present in LAM cells of both pulmonary LAM nodules and AMLs, indicating that the tumors originated from a common progenitor cell that was able to migrate or metastasize to and/or from either of these tissues (Sato et al., 2002). Although identical *TSC1* or *TSC2* mutations among tumor cells are not always observed in TSC-LAM and S-LAM patients, it is clear that LAM cells are mobile; markedly, LAM cells are able to infiltrate and repopulate healthy donor lungs following transplantation (Bittmann et al., 2003; Karbowniczek et al., 2003). They are also are able to metastasize between the lungs, kidneys, lymphatics, and uterus using mechanisms that do not fit the classic cancer metastasis paradigm. LAM cells can be found throughout the lymphatics in LAM patients, and their expression of VEGF-C and VEGF-D along with VEGF receptor-3 indicates their lymphangiogenic potential (Glasgow et al., 2012), indicating that the extensive network of lymphatics may serve as the primary niche for LAM cells and facilitates their spread.

LAM cells do not easily succumb to anoikis and can be found in the chylous fluid, urine, and circulating in the blood of LAM patients, a phenomenon that may be directly related to estrogen signaling (Crooks et al., 2004; Yu et al., 2009; Gu et al., 2013). Although these cells do not present themselves as malignant or highly proliferative compared to most cancer cells, their apparent robustness and infiltrative ability is certainly not benign.

A complete mechanistic model for LAM cell “metastasis” has yet to be confirmed; however, numerous studies have shown that LAM cells display increased motility and invasive properties *in vitro*. This invasive phenotype can be directly linked to loss-of-function mutations in either *TSC1* or *TSC2*, leading to pro-migratory cytoskeletal rearrangement and changes in focal adhesions. Primary LAM cells with TSC2 loss-of-function mutations display increased invasive and migratory properties as a result of RhoA GTPase-mediated cytoskeletal rearrangement and modulation of focal adhesions. RhoA GTPase is normally regulated by TSC1 when complexed with TSC2, and this invasive phenotype is mitigated upon TSC2 re-expression or TSC1 knockdown (Lamb et al., 2000; Goncharova et al., 2006). Perhaps contributing to the robustness of migratory LAM cells, dysfunctional TSC2 leads to a loss in membrane bound E-cadherin via a Rapamycin sensitive pathway. This, in turn, leads to reduced cell-cell adhesion, EMT, cell detachment, and resistance to anoikis (Barnes et al., 2010).

#### Molecular features and behavior of LAM cells parallel the neural crest

Clearly, dysfunction of either TSC1 or TSC2 will result in a cell that is primed to undergo EMT and migrate, a scenario that is reminiscent of neural crest delamination and migration during embryonic development. RhoA is critical in regulating the directionality of migrating neural crest cells, greatly affecting spatial-temporal patterning upon its loss (Rupp and Kulesa, 2007; Matthews et al., 2008). Cadherins play a crucial role during neurulation and help define the non-neural ectoderm and the neural plate (Taneyhill, 2008; Dady et al., 2012), the tissues whose interface from which neural crest cells delaminate and from which they migrate. Specifically, the transition from E-cadherin expression to N-cadherin plays a critical role in this process, and interestingly, a similar phenomenon is observed in LAM cells. As mentioned above, TSC2-deficiency results in internalization of E-cadherin leading to EMT; moreover, when LAM patient-derived cells are exposed to estrogen, E-cadherin expression is decreased and N-cadherin is upregulated, similar to the segregation of the germ layers during neurulation (Gu et al., 2013). Pairing well with the decrease in E-cadherin expression in TSC2-deficient cells, there is an upregulation in the expression of Snail, an important transcription factor involved in initiating EMT, migration, and neural crest specification (Cano et al., 2000; Aybar et al., 2003; Barnes et al., 2010). A second-hit mutation in *TSC2* therefore causes a change in the expression profile in LAM cells that resembles a neural crest progenitor undergoing migration and fate specification.

Adding fuel to the fire, LAM cells have been shown to excrete matrix metalloproteinases (MMPs), particularly MMP-2 and MMP-9, that likely contribute to their invasiveness and lead to the slow destruction of the lung parenchyma (Hayashi et al., 1997; Lee et al., 2010a). MMP2 and MMP9 are required in neural crest cells for their initial delamination and migration, and inhibition of these MMPs during development results in the complete halt of NC migration and differentiation (Monsonego-Ornan et al., 2012).

Extending this comparative train of thought, changes in TGF-ß signaling in LAM cells also relates directly to the NC and its lineages. Histopathological analysis of LAM cells, along with TSC2-null cell lines, show increased levels of TGF-ß and smooth muscle actin (SMA) (Zhe and Schuger, 2004; Barnes et al., 2010; Lee et al., 2010a). Although expression of TGF-ß and SMA is common to the EMT process, TGF-ß signaling is involved in NC-derived smooth muscle specification *in vivo* and is a common element of smooth muscle differentiation protocols of neural crest cells *in vitro* (Shah et al., 1996; Curchoe et al., 2010; Xie et al., 2013). The initiation of signaling cascades resulting in a smooth muscle-like cell fate may help explain the presence of spindle-like SMA+ proliferative cells in LAM nodules and angiomyolipomas. A loss of TSC2 function could destabilize a NC-derived cell, causing it to dedifferentiate and undergo EMT. This cell could then potentially migrate to a favorable niche (e.g., lung, kidney, lymphatics) and, due to increased TGF-ß signaling, differentiate into the smooth muscle cells and form the characteristic tumors associated with LAM.

The molecular signaling events that occur upon a second-hit mutation in TSC1 or TSC2 may act in some capacity to recapitulate the developmental processes of neural crest migration and specification. Such an orchestration may be enough to perhaps destabilize a dormant NCSC or a fully differentiated NC cell, whose epigenetic memory is already prone to EMT, migration, and cell motility. Indeed, with the proper stimulus, fully differentiated NC progeny display the ability to dedifferentiate and migrate *in vivo*, as well as return to a multipotent state when isolated and cultured *in vitro* (Real et al., 2005, 2006; Nagoshi et al., 2011; Dupin and Sommer, 2012).

The NC has already been implicated in the reactivation of EMT in fully differentiated and mature cells in the form of the metastatic cancers melanoma and neuroblastoma (Grimmer and Weiss, 2006; Shakhova, 2014). Furthermore, dedifferentiation and redifferentiation (EMT-MET switch) is a proposed mechanism of metastatic cancer supported by experimental evidence (Ocaña et al., 2012; Tsai et al., 2012). Neural crest cells have the unique ability to transdifferentiate during embryonic development from a neural ectodermal lineage to specialized cells that are ultimately considered to be mesodermal. This phenotypic plasticity is a unique quality of the neural crest, which clearly requires precise signaling and environmental cues during development for precise specification. Similar to the notion that a machine with many small moving parts is more likely to malfunction, neural crest-derived cells may be more prone to undergo EMT, migrate, and differentiate based on their inherent plasticity if given the proper cues.

#### Current models to investigate neural crest contribution to TSC

Few studies have directly investigated the hypothesis that *TSC1* or *TSC2* mutations in neural crest stem cells drive the development of non-CNS tumors in TSC, including LAM. Mice containing a conditional *TSC1* knockout allele crossed with mice expressing Cre driven by the *WNT1* promoter die prenatally, for unknown reasons, with no indication of renal or lung pathology (Kwiatkowski, 2010). No additional mouse or cell culture-based models of TSC1 or TSC2-deficiency in the neural crest lineage have been reported to date. The lack of clarity provided by existing mouse models, and the paucity of such models, regarding the development and progression of LAM in humans, heavily underscores the need for improved models that more closely recapitulate human pathology.

It is quite possible that the biology of TSC1- or TSC2-deficiency is substantially different in mouse vs. human NCCs, and that establishing the same models (e.g., Wnt1-cre conditional models of TSC1/2-deficiency) in a humanized system will allow us to generate accurate disease models. Alternatively, the lethality of the Wnt1-cre *TSC1* knockout mouse model may also serve as an indicator that homozygous *TSC* gene deletions within the neural crest lineage are not compatible with embryonic development and that TSC-like phenotypes will only be observed under conditions where second-hit mutations can be induced in a controlled manner within sub-populations of cells. Speaking to this, it is possible that different tumor types that are prevalent in TSC patients, as well as those that define LAM disease, are derived from TSC1- or TSC2-deficient cells of different neural crest subtypes. Identification of the NCC subtype(s) responsible for the development of LAM tumors may importantly result in less severe phenotypes and prolonged survival in animal models that should enable longer-term experiments to be performed than have been possible with current models. Additionally, this will inevitably result in disease phenotypes more specific to the LAM condition, allowing us to generate the most appropriate models possible.

### Strategies for greater understanding and improved treatment options in TSC and LAM

#### Theoretical model of neural crest contribution to disease heterogeneity

**Figure 2:**
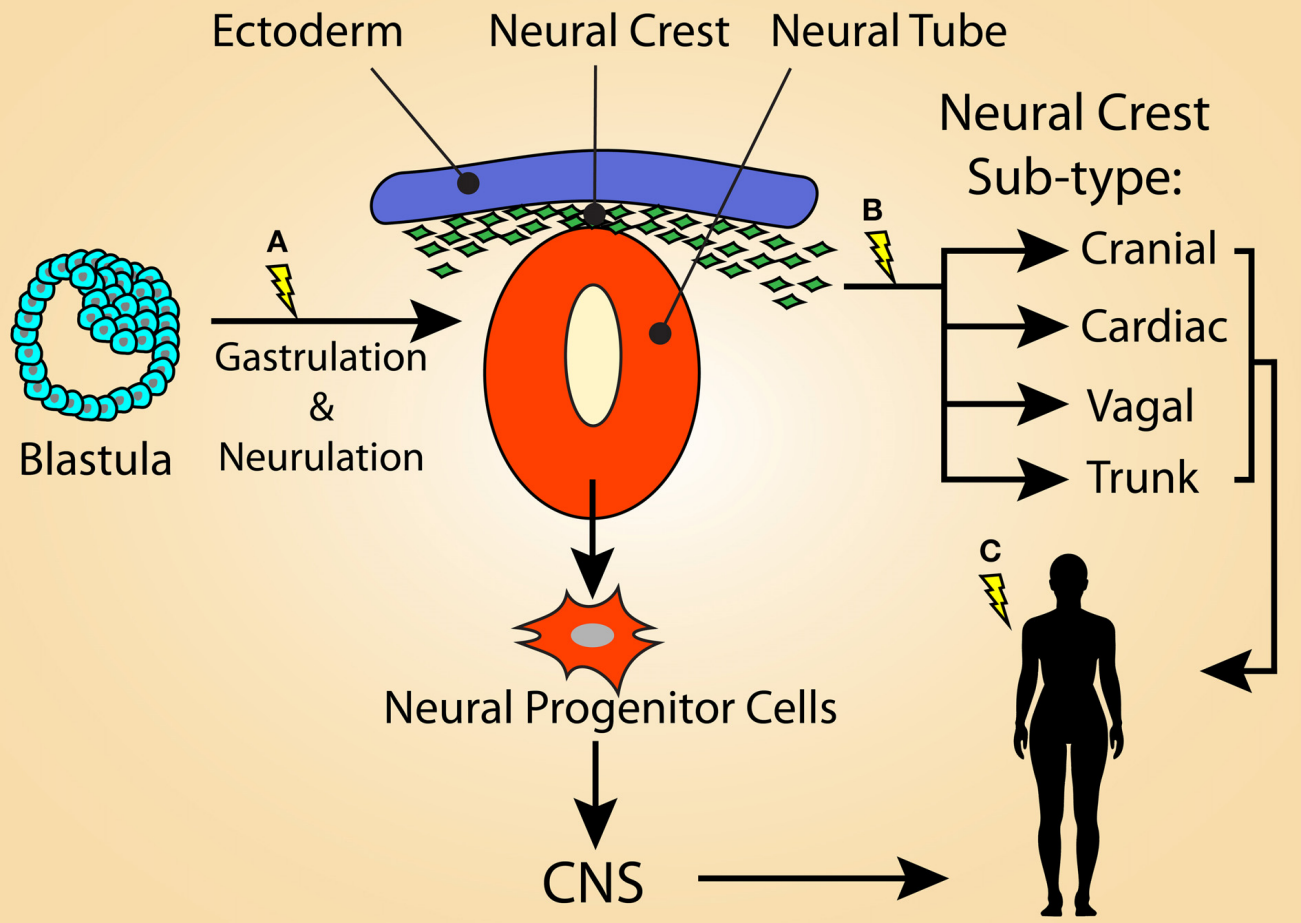
**Model illustrating that the variation of tumor heterogeneity in TSC may be related to the stage of development in which loss of function mutations in *TSC1* or *TSC2* occurs**. **(A)** For TSC patients with germline mutations, second-hit mutations may be acquired during early stages of embryogenesis, such as during gastrulation or neurulation. This would result in a large population of cells that carry the mutation and would lead to a severe TSC phenotype, possibly displaying the full spectrum of TSC manifestations. First-hit mutations at this time point would result in mosaicism. The affected cells could contribute not only to the neural crest lineage, but also the neural progenitor population of the neural tube, resulting in the CNS manifestations of TSC. **(B)** Second-hit mutations occurring in the emerging neural crest population likely results in many of the non-CNS symptoms of TSC, potentially including LAM. First-hit mutations at this time-point may result in S-LAM, as only the neural crest lineage would be vulnerable to second-hit mutations. It is possible that a sub-population of NCCs, such as the cardiac NC, is responsible for the more restricted lesions associated with S-LAM. **(C)** Alternatively, first- and/or second-hit mutations acquired within an adult NCC could also be responsible for driving LAM and some TSC-associated lesions (e.g., skin lesions).

While there is extensive correlative evidence for a potential role of the NC lineage as the cell of origin for LAM and many TSC manifestations, this hypothesis remains to be adequately tested experimentally. We present a model (Figure 2) whereby the diverse, heterogeneous tumor burden in TSC patients is dependent on when first- and second-hit mutations in *TSC1 or TSC2* occur during development and into adulthood. In this model, mutations that are carried in the germline or that are acquired during early stages of embryogenesis lead to a greater disease burden than those that occur at later time points. For instance, the majority of TSC patients have germline mutations, and thus all cell lineages, including both neural and neural crest progenitors, are vulnerable to the acquisition of second-hit mutations. Second-hit mutations may occur independently within the neural and neural crest lineages, or perhaps in a cell very early in development (i.e., before neurulation) (Figure 2A). In any case, TSC patients that carry a germline *TSC1* or *TSC2* mutation are likely to acquire wide-spread and severe disease manifestations due to the diverse pool of progeny susceptible to acquiring second-hit mutations (i.e., both the NCC and NSC lineages).

Similarly, a first-hit mutation acquired at a time point as early as neurulation (Figure 2A) would give rise to sporadic TSC, explaining the genotypic mosaicism observed in some patients. In this scenario, the severity of the resulting disease spectrum depends on the distribution pattern of “first-hit” cells into downstream lineages as well as in which cell populations second-hit *TSC* gene mutation(s) are acquired. Here, both the NCC and NSC lineages are vulnerable to being infiltrated by *TSC* mutant cells, however mosaicism may lead to reduced disease severity by restricting the number of cells or the cell lineages that carry *TSC* mutations.

Following this model further, the more limited disease manifestations observed in S-LAM, and potentially milder forms of TSC, would originate from a primary *TSC* gene mutation in a much more restricted population limited to the NC lineage. This could occur either during embryogenesis within a common or sub-type restricted neural crest progenitor cell (Figure 2B) or in adulthood (Figure 2C), affecting NCSCs or NC-derived cells, such as melanocytes or SKPs. The former possibility could foreseeably occur either via mosaicism within the broader NC lineage, in which only certain NC subtypes carry enough of a mutational burden to drive the development of a lesion, or restriction of first- and/or second-hit mutations within a functionally defined sub-population of NC. A likely candidate is the cardiac NC lineage due to its involvement in development of the aorticopulmonary septum. In either scenario, only cells that were derived from NCCs during development or NCSCs that persist in adult tissues are vulnerable to a second-hit mutation later in life that may endow them with the ability to drive the formation of TSC and LAM lesions. This highlights the possibility that the heterogeneity within TSC and S-LAM patients is predominantly dictated by the cells that carry a first-hit *TSC* gene mutation and, consequently, the resulting progeny left vulnerable to acquiring a second-hit mutation.

As mosaicism within the NC lineage may potentially lead to the more restricted phenotypes associated with LAM, so too may gonadal or somatic mosaicism lead to varying degrees of phenotypic severity in TSC patients, as has been observed clinically (Rose et al., 1999; Verhoef et al., 1999; Qin et al., 2010b; Boronat et al., 2014a,b). This type of model is therefore likely to underlie phenotypic differences between TSC vs. LAM alone, as well as the heterogeneity in disease features observed in both TSC and LAM patient cohorts themselves. Thus, a *TSC1* or *TSC2* mutation at an earlier developmental stage has the potential to result in a more complete spectrum of TSC phenotypes, while a later mutation will lead to a more restricted pathological potential, resulting in limited TSC phenotypes or LAM only.

#### Current treatment options for TSC and LAM

While double lung transplant is currently the gold standard treatment option for LAM, this approach is not possible in all patients; additionally, LAM lesions have been shown to recur in the donor lung in some patients (Bittmann et al., 2003; Karbowniczek et al., 2003). Thus, transplantation does not address the underlying mechanisms that drive the development of LAM cells and associated lesions. The mTOR-specific inhibitor Rapamycin is a promising molecular intervention currently under intensive investigation as a potential treatment for LAM and other manifestations in TSC. Rapamycin has proven clinically effective for the treatment of most TSC-associated lesions (Franz et al., 2006; Bissler et al., 2008; Krueger et al., 2010; Micozkadioglu et al., 2010; Casanova et al., 2011; Dabora et al., 2011; DeKlotz et al., 2011; Tiberio et al., 2011; Canpolat et al., 2014; Park et al., 2014), and in the context of LAM can lead to reduction in the size of AMLs, provide stabilization of lung function, and a marked improvement in quality of life measures (McCormack et al., 2011).

A major caveat with this approach, however, is that Rapamycin is tumoristatic rather than tumoricidal. Thus, disease progression resumes once Rapamycin is withdrawn. Therefore, patients must remain on the drug permanently to provide sustained treatment. Long-term Rapamycin treatment is also not without potential challenges, as there is concern that LAM tumors, as well as other TSC lesions, may become refractory to Rapamycin over an extended time period. Additionally, while Rapamycin treatment can reduce mTOR signaling, it may also have the undesirable effect of promoting the survival of TSC1 or TSC2-null cells (reviewed in Henske and McCormack, 2012). In this regard, one of the key downstream processes controlled by mTORC1 signaling is autophagy (Laplante and Sabatini, 2012), a key cellular survival mechanism, and inhibition of autophagy is a typical consequence of TSC1- or TSC2-deficiency (Crino et al., 2006; Parkhitko et al., 2011; Crino and Tsai, 2012; Henske and McCormack, 2012). Thus, while Rapamycin appears to generally inhibit the growth of TSC1- or TSC2-deficient cells, it may concurrently promote their survival by inducing autophagy. Rapamycin has also been shown to induce expression of pro-survival micro-RNAs (Trindade et al., 2013). Thus, Rapamycin treatment may therefore result in a precarious situation whereby the growth and proliferation of TSC1- or TSC2-deficient cells is halted, but the cells remain alive *in situ*, allowing them to re-expand once treatment is stopped or an alternative mTOR signaling pathway is triggered in the extensive mTOR signaling network to bypass mTORC1 inhibition.

As long-term Rapamycin treatment carries a significant risk of acquired treatment resistance and adverse side-effects (McCormack et al., 2011), additional therapeutic options are needed to provide life-long treatment options to TSC and LAM patients. Importantly, a number of proteins thought to be important in LAM pathogenesis are unaffected by Rapamycin treatment in LAM cells, including MMP2 (Lee et al., 2010b). Due to this fact and observations that alternative pathways are activated by Rapamycin treatment that promote cell survival, there is a strong indication for the development of alternative or combination therapies. Thus, the development of new therapeutic approaches is currently of the highest priority within the LAM and TSC fields. In concert with this, the development of effective models to aid in the identification and testing of potentially effective therapeutic interventions must also be at the top of the list.

Identification of the cell of origin for LAM and other TSC-associated tumors is imperative for the development of more effective, long-lasting treatment strategies. Only by inducing first- and second-hit mutations in the proper cell of origin, and in the proper proportion of that population, will we be able to fully understand and therapeutically target the molecular mechanisms that drive the initiation and progression of distinct TSC lesions. mTOR activation can lead to different primary phenotypes in distinct cell types, such as precocious differentiation in the CNS (Hartman et al., 2013), increased cycling of hematopoietic stem cells (Gan et al., 2008), and exit of satellite cells from quiescence upon injury (Rodgers et al., 2014). Therefore, different TSC-associated lesions may require different targeted therapeutic approaches. Furthermore, the downstream targets of mTOR are diverse and affect multiple cellular signaling pathways, and mTOR participates in a number of feed-back signaling mechanisms (Laplante and Sabatini, 2012). Due to this complexity, truly effective treatment approaches are likely to require combination therapies in which multiple signaling nodes are targeted, and the most promising approaches may well be different for distinct tumor types.

#### A need for humanized disease models

The TSC and LAM fields have benefited significantly from cell culture-based and rodent models of TSC1- or TSC2-deficiency; however, these models carry a number of caveats. Rodent models of spontaneous (e.g., the Eker rat) or genetically engineered *TSC* gene deficiency, and cells cultured from these animals (e.g., ELT3 cells, isolated fibroblasts), have offered important insights into the basic molecular and cellular phenotypes caused by *TSC* ablation and have provided avenues for experimental drug testing. However, these models do not fully recapitulate the tumor spectrum or phenotype of many TSC lesions or their derivative cells, including LAM. While these animal models exhibit a high penetrance of renal cystadenomas and liver hemangiomas with age, and a partial penetrance (about 30%) of lung adenomas, these tumors do not recapitulate the phenotypes of those observed in human TSC patients (Kobayashi et al., 1999, 2001; Onda et al., 1999; Kwiatkowski et al., 2002; Kwiatkowski, 2010). Therefore, they cannot be used to understand the pathophysiology of the most clinically significant manifestations of TSC, nor as accurate pre-clinical models for drug testing to treat or eliminate these lesions.

Presently, there is a lack of humanized-models of TSC and LAM. Primary TSC1- or TSC2-deficient cells from TSC or LAM patients unfortunately cannot be efficiently propagated in culture without viral transformation, and even then some elements of LAM cell identity observed *in vivo* are not recapitulated in culture. Additionally, patient-derived cells lack proper control cell lines, and induction of TSC1- or TSC2-deficiency (disruption of both alleles or a high-degree mRNA knockdown), as is the case for most of the existing models, bypasses mechanisms that lead to second-hit mutations. As this important feature of TSC and LAM tumor pathogenesis is absent, these systems cannot be used to understand the processes leading to tumor induction and subsequent disease progression. Thus, to accurately model TSC and LAM it will be essential to generate or obtain human cell lines in which first- and second-hit *TSC* gene mutations can be induced in a controlled manner, and in which cellular phenotypes can be clearly assessed following differentiation into putative cell of origin lineages. Identification of the cell types of origin for TSC and LAM lesions is an essential step toward the development of accurate pre-clinical humanized disease models. Such models will not only allow for a more relevant understanding of the mechanisms that drive disease, but will also greatly improve therapeutic options for patients, making personalized therapeutics a potentially viable option for TSC.

#### Approaches for generating humanized disease models

While it is clear from mouse models that it is at least possible to induce TSC-like brain lesions by manipulating mTOR signaling in the NSC lineage, no humanized models exist to test whether the same events are indeed responsible for driving CNS pathologies in humans. Furthermore, while a NC origin for non-CNS lesions in TSC seems intuitive, direct experimental evidence testing this hypothesis in human cells is needed. To perform these pivotal experiments, we need better disease models. First, we need to generate human cell lines that will recapitulate phenotypes observed in distinct TSC and LAM lesions, and can potentially give rise to these lesions *in vitro* and in *in vivo* mouse models, following controlled induction of TSC1- or TSC2-deficiency. Second, the behavior of the cells that comprise TSC lesions appears to be highly dependent on interactions with their tissue microenvironment. Thus, proper disease models should incorporate *in vivo* and *in vitro* culture-based approaches that closely recapitulate the natural, *in situ* environment in which TSC and LAM lesions normally develop.

One promising approach for the generation of humanized models in TSC is to couple genetic engineering to induce TSC1/2-deficiency with embryonic stem cell (ESC) or patient-derived induced pluripotent stem cell (iPSC) technology. ESCs and iPSCs are pluripotent stem cell populations that have the capacity to differentiate into any cell type found in the body, including candidate cell types of origin for TSC and LAM lesions. Furthermore, iPSCs importantly offer an avenue for patient-specific modeling, as it allows fully differentiated cells from TSC and LAM patients harboring *TSC* gene mutations to be reprogrammed to an ESC-like state, enabling subsequent directed differentiation into putative cell of origin populations.

Directed differentiation of human ESCs or iPSCs into neural and neural crest lineages *in vitro* and *in vivo*, coupled with the ability to inducibly knock-out or knock-down TSC1 or TSC2 at different stages of differentiation, will directly allow cell of origin theories to be tested. This will also allow for the establishment of human cell lines that reflect TSC and LAM cell phenotypes and, following *in vivo* injection of TSC1/2-deficient cell lines, the generation of humanized animal models of LAM and TSC lesions. Subsequently, growth and differentiation of these cell lines in not only 2-dimensional, but also 3-dimensional culture conditions, as well as injection into immune-compromised mice, will enable the behavior of these cells to be studied in environments that more closely recapitulate the *in vivo* environment of distinct TSC and LAM lesions.

A second, highly promising approach that would offer opportunities for patient-centered discovery and treatment is to use endogenous sources of NC-like stem cells resident within adult tissues, such as SKPs, to generate TSC1- or TSC2-deficient lines. SKPs reside in a location that is highly accessible for tissue extraction and subsequent NC cell isolation, thus they may be a particularly useful cell population to allow for the development of patient-specific models. Development of models in which a patient's own cells can be used as the source material to follow disease initiation and progression will importantly allow us to understand not only the basic mechanisms of TSC and LAM tumor formation, but also what drives the heterogeneous phenotypes that are often observed between different patients.

## Conclusion

The TSC and LAM research communities have moved at an impressive pace in the last two decades. Since discovering the *TSC2* gene in 1993 (European Chromosome 16 Tuberous Sclerosis Consortium, 1993), investigative research efforts have quickly produced a plethora of experimental data leading to the development of the first generation of therapeutics for TSC and LAM. However, due to the pitfalls associated with the largely tumoristatic action of Rapamycin and closely associated “rapalogs,” it is apparent that a wider array of advanced treatment options is necessary to combat these multisystem disorders. Current disease models of TSC and LAM have been invaluable in providing insight into molecular disease pathology, provided a basis for clinical trials of rapalogs and other potential therapeutics, and has supported the investigation of neural stem cell of origin theories for TSC CNS manifestations. However, these same disease models have their limitations and do not fully recapitulate the nature or progression of TSC and LAM observed in humans.

There is sufficient evidence to suggest that the NC lineage is integrally involved in TSC and LAM tumor heterogeneity; however, this notion has largely been limited to discussion due to a lack of an effective means to study such theories. Currently, there are no humanized models of TSC and LAM that provide a framework for investigating this theory, nor to investigate the influence of *TSC* gene mutations acquired during early development on disease heterogeneity. Yet, with the current advances in tissue culture methods, stem cell-based disease modeling, and the advent of iPSC technology, this will likely change in quick order. Using humanized models to identify the cell of origin for LAM and other TSC tumors is crucial in identifying and targeting the specific molecular mechanisms responsible for distinct TSC and LAM lesions, and will help pave the way for the next generation of TSC and LAM therapeutics.

### Conflict of interest statement

The authors declare that the research was conducted in the absence of any commercial or financial relationships that could be construed as a potential conflict of interest.
